# Identification and Antibiotic Resistance of Isolates from Poultry Meat and Poultry Meat By-Products Exhibiting Characteristic *Salmonella* Morphology on Chromogenic Agar

**DOI:** 10.3390/antibiotics14060540

**Published:** 2025-05-24

**Authors:** Sarah Panera-Martínez, Cristina Rodríguez-Melcón, Camino González-Machado, Carlos Alonso-Calleja, Rosa Capita

**Affiliations:** 1 Department of Food Hygiene and Technology, Veterinary Faculty, University of León, E-24071 León, Spain; 2 Institute of Food Science and Technology, University of León, E-24071 León, Spain

**Keywords:** *Salmonella*, poultry, chromogenic agar, selectivity, false positives, antibiotic resistance

## Abstract

**Background/Objectives**: The main objective of this research work was to identify and determine the antibiotic resistance of the false-positive isolates on chromogenic agar when analyzing *Salmonella* in chicken meat. **Methods**: A total of 234 samples of chicken meat (carcasses, cuts and preparations) were studied using buffered peptone water for primary enrichment, Rappaport–Vassiliadis soy broth for secondary enrichment and *Salmonella* Chromogen Agar Set as a selective solid medium. Colonies with a morphology characteristic of *Salmonella* (one isolate per sample) were identified by matrix-assisted laser desorption ionization and time-of-flight mass spectrometry (MALDI-TOF). **Results**: Colonies with a characteristic morphology of *Salmonella* were detected in 71 samples. Only five isolates (7.0% of the total) corresponded to the genus *Salmonella*. Other genera detected were *Hafnia* (three isolates; 4.2% of the total), *Escherichia* (22; 31.0%), *Klebsiella* (19; 26.8%), *Proteus* (6; 8.5%) and *Pseudomonas* (16; 22.5%). The 66 isolates of these last five genera were tested for susceptibility to a panel of 42 antibiotics of clinical importance by disc diffusion. All isolates presented multiple resistances, to between 4 and 29 antibiotics, all of them having a multi drug-resistant (MDR) phenotype except for one *Pseudomonas* strain, with an extensively drug-resistant (XDR) phenotype. **Conclusions**: These results highlight the low selectivity of this method, with the specific culture media under test, for the detection of *Salmonella* in poultry meat. The considerable prevalence of antibiotic resistance observed suggests a need to improve control measures throughout the poultry meat production chain to prevent this food from becoming a reservoir of bacteria with resistance to multiple antibiotics.

## 1. Introduction

Enterobacteriaceae comprise a very diverse family of Gram-negative bacteria widely distributed in the environment, whose main habitat is the intestinal tract of humans and animals [1]. The slaughtering, gutting and processing procedures can promote contamination of poultry meat, which is a frequent vehicle for diseases caused by Enterobacteriaceae [2]. Despite this, its satisfactory nutritional properties and ease of cooking make it a highly demanded food [3], with the forecast that by 2032 poultry meat will represent 41.0% of the animal protein consumed around the world [4].

Some of the genera of Enterobacteriaceae frequently associated with poultry meat and their derived products are *Escherichia*, *Serratia*, *Hafnia*, *Salmonella*, *Klebsiella*, *Yersinia* and *Proteus* [5]. The levels of Enterobacteriaceae in such meat are used as indicators of its microbiological quality, as well as the degree of adherence to good hygienic practices throughout its production and distribution chain [3]. For the determination of Enterobacteriaceae in foods, techniques based on the incubation and cultivation of samples in specific media for each microbial group are usually employed [6].

The ISO 6579-1:2017 [7] standard is an official method for the detection, enumeration and serotyping of *Salmonella* spp. in food samples and consists of the enrichment of the samples in liquid culture media and subsequent plating onto selective solid media. Selective media for *Salmonella* spp. are designed to allow the growth of this microorganism while inhibiting the proliferation of others. However, different bacteria can form colonies of similar morphology and color to those of *Salmonella*, leading to what can be termed false positives [8].

Many species of Enterobacteriaceae are not pathogenic to humans or, if so, they simply cause mild gastrointestinal infections that generally resolve spontaneously within a few days without the need for antibiotic treatment [9]. However, some Enterobacteriaceae can act as a reservoir of antibiotic resistance genes, which can be horizontally transferred to other bacterial species or genera with which they share a habitat [10]. This fact is especially worrying, since in recent years an increase in resistance to antibiotics has been observed in various groups of bacteria, including Enterobacteriaceae [11], and it is feared that soon infections caused by bacteria resistant to multiple antibiotics will become consolidated as the leading cause of death worldwide [12]. In addition to the implications of antibiotic resistance for public health, infections caused by resistant bacteria are associated with higher costs for health systems because of the greater probability of hospital admission and longer stays [13].

To guarantee the safety of poultry meat, it is essential to have adequate methods for detecting any pathogenic microorganisms present in it. The objective of the research work being reported here was to identify the colonies with a morphology characteristic of *Salmonella* formed on *Salmonella* Chromogen Agar Set after primary enrichment in buffered peptone water and secondary enrichment in Rappaport–Vassiliadis soy broth, with the aim of determining the false-positive results and evaluating the selectivity of this isolation method. In addition, the antibiotic resistance of false-positive strains was determined.

## 2. Results

### 2.1. Identification of Colonies with Characteristic Morphology of Salmonella

In 71 of the 234 samples tested, colonies with the typical morphology and color of *Salmonella* were observed on the chromogenic medium and a colony of each sample was taken. These colonies were detected and isolated from 32 (61.5%) of the 52 chicken carcasses obtained in slaughterhouses, from 22 (42.3%) of the 52 chicken cuts acquired in slaughterhouses and from 17 (13.0%) of the 130 chicken cuts and chicken meat preparations purchased in butcher’s shops.

**Figure 1:**
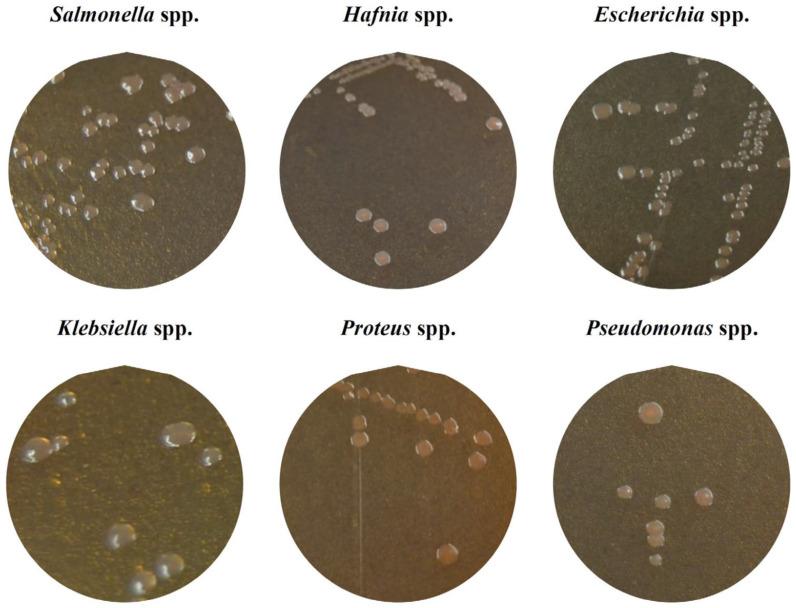
Appearance of Enterobacteriaceae colonies selected from chromogenic culture medium for detecting *Salmonella* spp. (*Salmonella* Chromogen Agar Set, Sigma-Aldrich, St. Louis, MO, USA).

**Figure 2:**
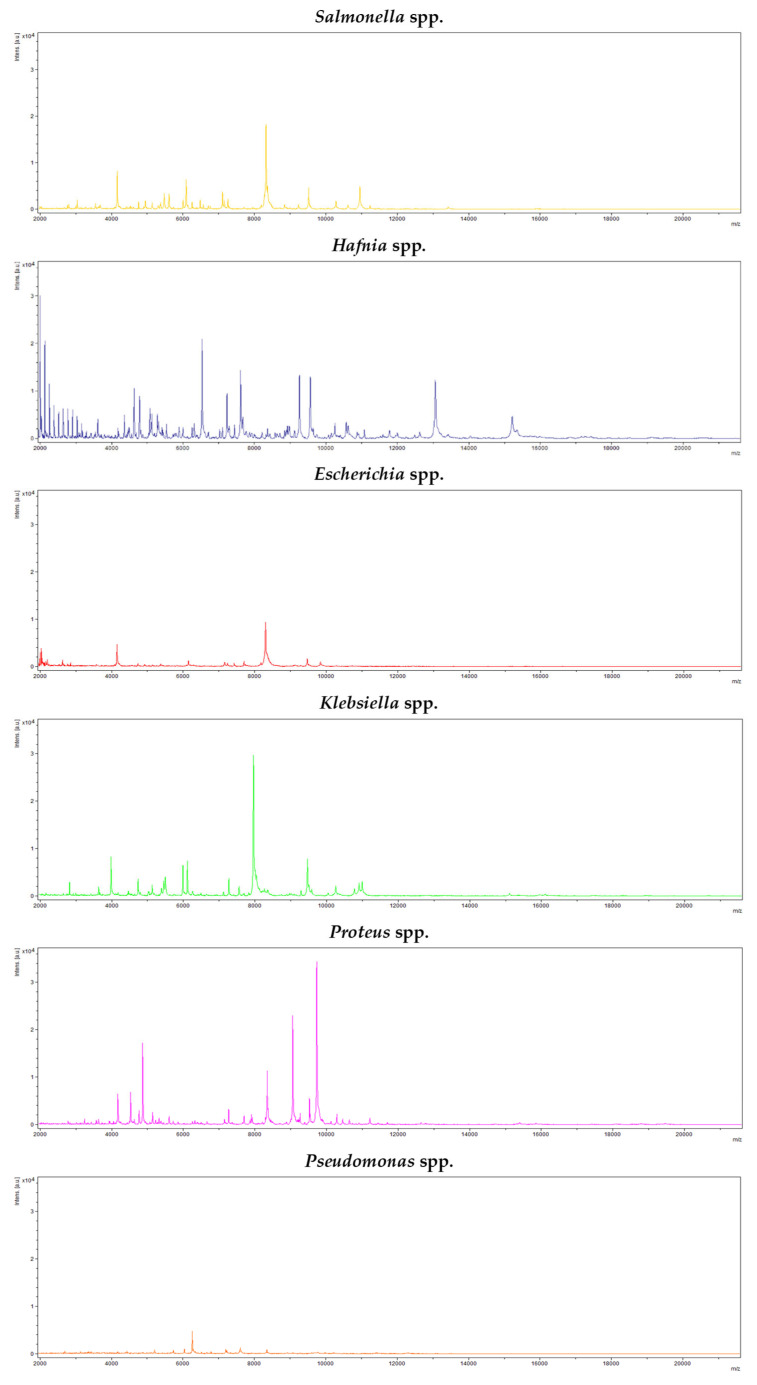
Spectra obtained for each genus of bacteria identified by MALDI-TOF: *Salmonella*, *Hafnia*, *Escherichia*, *Klebsiella*, *Proteus* and *Pseudomonas*.

Identifications carried out using matrix-assisted laser desorption ionization and time-of-flight mass spectrometry (MALDI-TOF) revealed the presence of six bacterial genera: *Salmonella* was found in five isolates (7.0% of the isolates), *Hafnia* in 3 (4.2%), *Escherichia* in 22 (31.0%), *Klebsiella* in 19 (26.8%), *Proteus* in 6 (8.5%) and *Pseudomonas* in 16 (22.5%). Thus, 93.0% of the colonies with a morphology typical of *Salmonella* turned out to be false positives. Figure 1 shows the appearance of the colonies of each genus (*Salmonella*, *Hafnia*, *Escherichia*, *Klebsiella*, *Proteus* and *Pseudomonas*) on the chromogenic medium and Figure 2 shows an example of the spectra obtained with the MALDI-TOF system MS Biotyper for each of the genera in question.

**Figure 3:**
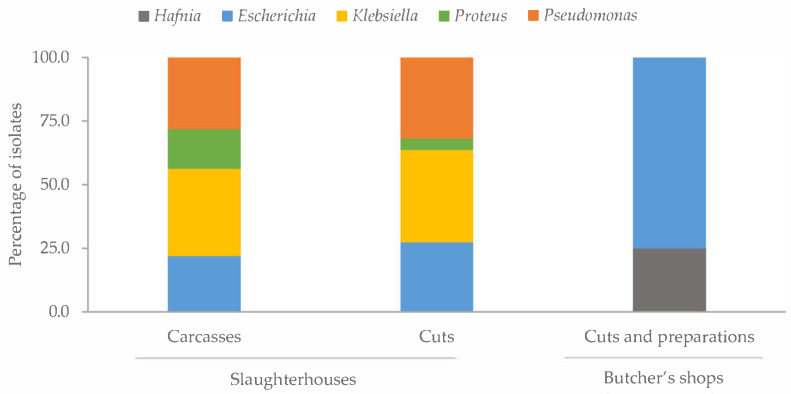
Percentage of strains identified by MALDI-TOF as from the genera *Hafnia*, *Escherichia*, *Klebsiella*, *Proteus* and *Pseudomonas* in the three types of samples studied. These were whole chicken carcasses and cuts from abattoirs and chicken cuts and preparations from butcher’s shops.

All the *Salmonella* colonies were isolated from samples acquired in butcher’s shops (chicken cuts and chicken meat preparations). The distribution of the remaining genera detected varied according to the type of sample analyzed (Figure 3). It should be noted that in the cuts and preparations from butcher’s shops, only the genera *Hafnia* and *Escherichia* were found, the latter being the predominant genus, accounting for 75.0% of the strains isolated from these samples. The prevalence of the different bacterial genera was similar in the whole chicken carcasses and cuts from slaughterhouses, except for *Proteus*, which had a higher prevalence in the carcasses (*p* < 0.05).

### 2.2. Antibiotic Susceptibility

The 66 colonies isolated from the chromogenic medium that were not identified as *Salmonella* (false positives) were tested against a panel of 42 antibiotics of clinical importance. Taking together all the isolates and all the antibiotics used, 2772 determinations were performed (66 × 42 = 2772). In these, 37.7% showed resistance, 10.6% reduced susceptibility and 51.7% susceptibility. Analysis of these data by antibiotic involved made it clear that more than 95.0% of the strains studied were susceptible to AK, FOX, IPM and CT, but resistant to TEC and E. Furthermore, between 70.0% and 95.0% of the strains showed susceptibility to CN, MEM, TZP, C, FOS and TGC and resistance to RD and P. It is worth highlighting that considerable percentages of strains had reduced susceptibility to N (37.9%), S (51.5%) and TEM (69.7%). The remaining antibiotics showed variable resistance percentages, ranging between 12.1% for KZ and 62.1% for NA and S3.

**Figure 4:**
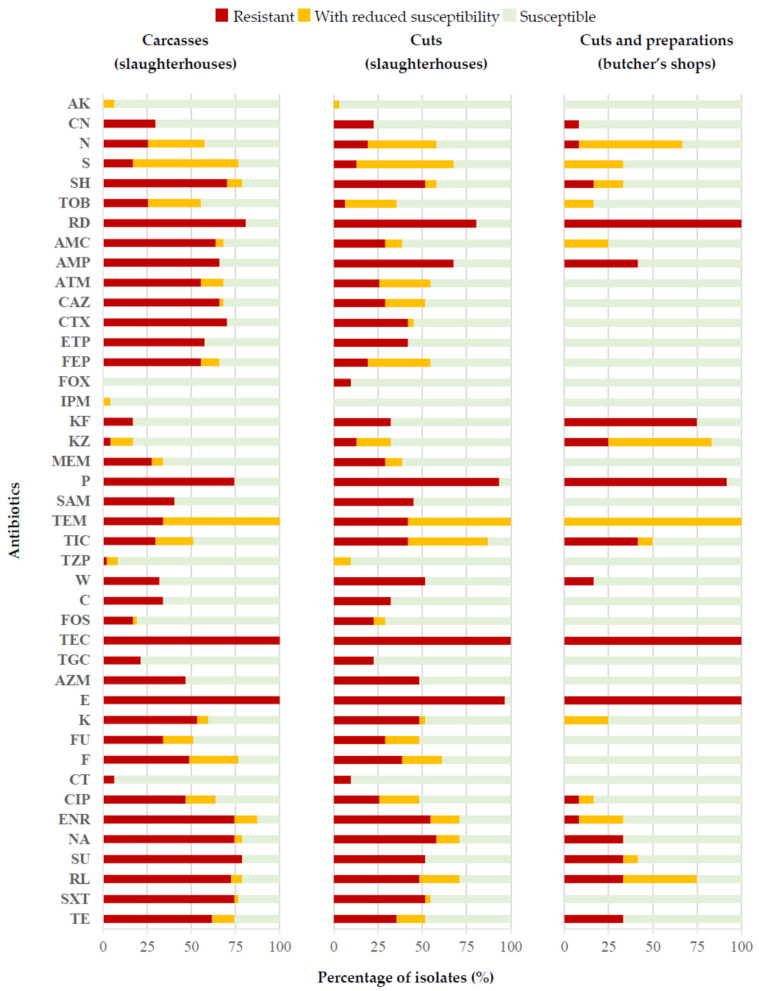
Percentage of Enterobacteriaceae and *Pseudomonas* strains showing resistance, reduced susceptibility or susceptibility to each antibiotic tested, relative to the origin of samples. AK (amikacin, 30 μg), CN (gentamicin, 10 μg), K (kanamycin, 30 μg), N (neomycin, 30 μg), S (streptomycin, 10 μg), SH (spectinomycin, 100 μg), TOB (tobramycin, 10 μg), RD (rifampin, 5 μg), AMC (amoxycillin/clavulanic acid, 30 μg), SAM (ampicillin/sulbactam, 20 μg), AMP (ampicillin, 10 μg), P (penicillin G, 10 μg), TEM (temocillin, 30 μg), TIC (ticarcillin, 75 μg), TZP (piperacillin/tazobactam, 110 μg), CAZ (ceftazidime, 30 μg), CTX (cefotaxime, 30 μg), FEP (cefepime, 30 μg), FOX (cefoxitin, 30 μg), KF (cefalothin, 30 μg), KZ (cefazolin, 30 μg), ETP (ertapenem, 10 μg), IPM (imipenem, 10 μg), MEM (meropenem, 10 μg), ATM (aztreonam, 30 μg), C (chloramphenicol, 30 μg), FOS (fosfomycin, 50 μg), TEC (teicoplanin, 30 μg), TGC (tigecycline, 15 μg), AZM (azithromycin, 15 μg), E (erythromycin, 15 μg), F (nitrofurantoin, 300 μg), FR (furazolidone, 100 μg), CT (colistin, 10 μg), CIP (ciprofloxacin, 5 μg), ENR (enrofloxacin, 5 μg), NA (nalidixic acid, 30 μg), S3 (sulfonamide, 300 μg), RL (sulphamethoxazole, 25 μg), SXT (sulphamethoxazole/trimethoprim, 25 μg), W (trimethoprim, 5 μg) and TE (tetracycline, 30 μg).

Figure 4 shows the data obtained relative to the type of sample involved, whether whole chickens or cuts from abattoirs, or cuts or meat preparations from butcher’s shops, with significant differences (*p* < 0.05) being observed between compounds. Thus, the strains isolated from preparations and cuts obtained from butcher’s shops were those presenting the lowest percentages of resistance to almost all the compounds. One exception was KF, an antibiotic for which the strains isolated from this type of sample reached a value of 75.0% of resistance, whilst in carcasses and cuts obtained from slaughterhouses the corresponding figures were between 17.0% and 32.3%. On the other hand, it was striking that the strains from carcasses had the highest percentages of resistance to several of the beta-lactam antibiotics studied (AMC, ATM, CAZ, CTX, ETP and FEP), with values ranging between 55.3% and 70.2% for the strains from this type of sample, whereas they fell between 0.0% and 41.9% in the rest of the strains, from cuts obtained from abattoirs, or cuts and preparations from butcher’s shops. This was also the case for other antibiotics (SH, TOB, CIP, ENR, S3, RL, SXT and TE) with the highest percentages of resistance noted in strains isolated from whole chickens acquired from slaughterhouses. It should also be noted that the highest percentages of resistance observed in the case of antibiotics P, W and CT were seen in strains isolated from cuts obtained from abattoirs.

**Figure 5:**
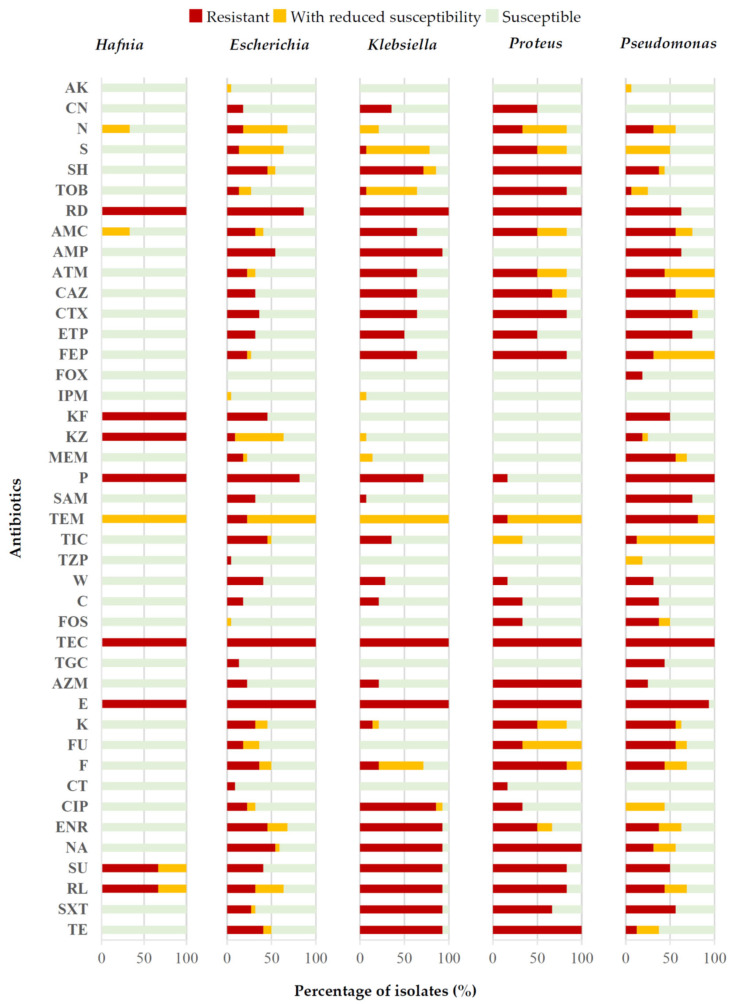
Percentage of isolates showing resistance, reduced susceptibility or susceptibility to each antibiotic tested, by genus of bacteria. For additional interpretation see Figure 4.

Figure 5 shows the antibiotic resistance data ordered by genus of bacteria, differences also existing (*p* < 0.05) between compounds. In this case, it may be seen that strains of the genus *Hafnia* presented the lowest percentages of resistance to most of the antibiotics tested, except for RD, KF, KZ and P. The *Klebsiella* strains showed the highest percentages of resistance to some beta-lactam antibiotics (AMP and ATM), quinolones (CIP and ENR) and sulphonamides (SXT). Further, every strain of *Proteus* was resistant to AZM, whilst in all the other genera the percentage of isolates resistant to this compound did not exceed 25.0%. The isolates identified as *Proteus* also presented a high prevalence of resistance (more than 80.0% of the strains) to SH, TOB, CTX, FEP, F and TE. It is noteworthy that a large percentage (more than 75.0%) of *Pseudomonas* strains showed resistance to ETP, SAM and TEM. In the case of the remaining antibiotics, the percentages of resistance were more homogeneous from one genus of bacteria to another.

### 2.3. Antibiotic Resistance Patterns

The 66 isolates tested showed resistance to between 4 and 29 antibiotics belonging to at least three different categories. An average value of 15.83 ± 7.02 resistances per isolate was recorded, with this rising to 20.29 ± 6.82 if resistance and reduced susceptibility were taken together. Table 1 shows the average data for resistance, reduced susceptibility or susceptibility for the isolates, relative to the type of sample analyzed and the genus considered.

**Figure 6:**
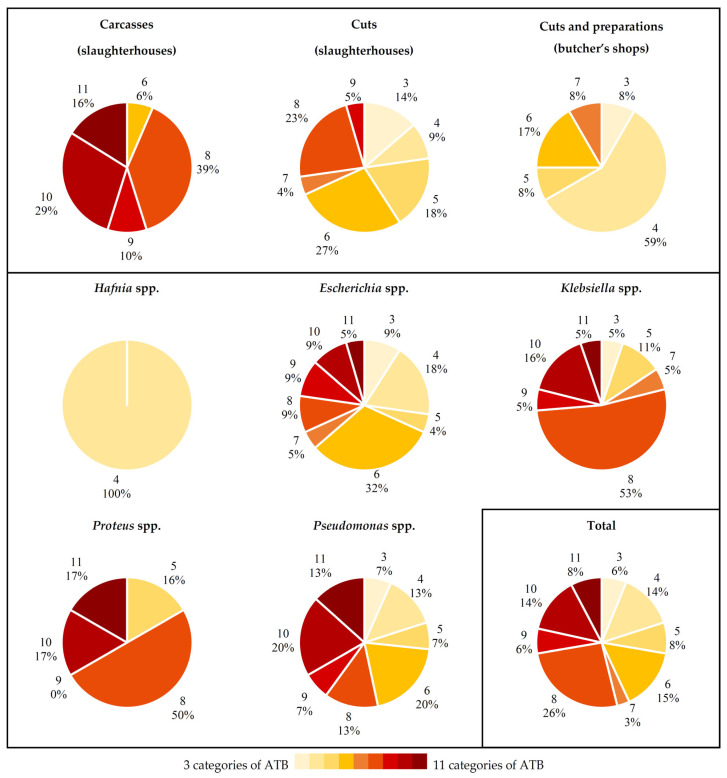
Percentages of strains with an MDR phenotype. These were resistant to between 4 and 29 of the 42 antibiotics evaluated, belonging to between 3 and 11 categories. The results are shown by type of sample investigated (upper graph; carcasses from slaughterhouses, cuts from slaughterhouses and cuts and preparations from butcher’s shops), by bacterial genus (lower graph; *Hafnia*, *Escherichia*, *Klebsiella*, *Proteus* and *Pseudomonas*) and overall (bottom right graph; total). ATB, antibiotics. The graphs indicate the number of categories of antibiotics to which there was resistance, and the percentage of strains noted in each case.

One strain of *Pseudomonas* spp. isolated from whole chickens presented an XDR phenotype, with resistance to 28 antibiotics, from 12 out of the 13 categories tested. The rest of the isolates studied presented an MDR phenotype, with resistance to between 4 and 29 antibiotics, from between 3 and 11 different categories (Figure 6).

If the type of sample from which each strain came is considered, it may be seen that the strains isolated from carcasses obtained from slaughterhouses had resistances to between 6 and 11 of the categories of antibiotics tested. In the other two groups of samples the corresponding figures ranged between 3 and 9, in the case of cuts from abattoirs, or between 3 and 7, for cuts and meat preparations acquired in butcher’s shops. Furthermore, it is striking that a considerable percentage (59.0%) of strains in this last group of samples showed resistance to four categories of antibiotics.

Consideration of the data by bacterial genus reveals that the *Hafnia* isolates presented resistance to only four different categories of antimicrobials (rifamycins, penicillins, cephalosporins and macrolides). For all the other genera identified, the results were more variable, although more than 50.0% of the *Proteus* and *Klebsiella* isolates showed resistance to 8 of the 13 categories of antibiotics tested. On the other hand, strains of the genera *Escherichia* and *Pseudomonas* showed more homogeneous percentages of resistance to the various groups of antimicrobials.

**Figure 7:**
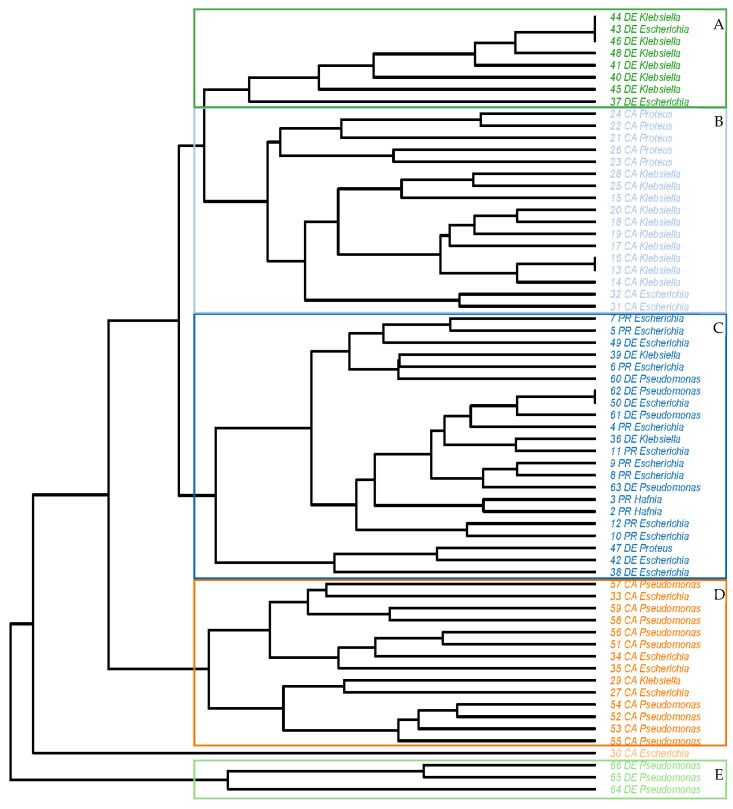
Tree diagram of antibiotic resistance profiles of the 66 strains investigated. This graph was drawn up based on an unweighted pair group method with arithmetic mean (UPGMA) analysis and Euclidean distances derived from co-ordinates. CA, carcasses obtained from slaughterhouses; DE, cuts acquired from slaughterhouses; PR, meat preparations and cuts obtained from butcher’s shops.

Considering the antibiotic resistance profiles observed for each strain studied, it yields the tree-diagram of Figure 7. The strains fall into five clusters and an individual isolate. Four of the clusters are dependent upon the type of sample from which the isolates came: two groupings are formed of strains isolated from whole chickens obtained from abattoirs and a further two of isolates originating from cuts acquired from that same source. The first cluster (A) is composed of eight isolates, from the genus *Klebsiella* (six isolates) and *Escherichia* (two). Cluster B comprises isolates of *Proteus*, *Klebsiella* and *Escherichia* from carcasses obtained from slaughterhouses, with the greatest similarities being found between strains belonging to the same bacterial genus. Group C is the most heterogeneous, with strains of the five genera studied, isolated both from cuts and chicken preparations from butcher’s shops and from cuts from abattoirs. Group D is made up of isolates of *Escherichia*, *Klebsiella* and *Pseudomonas* from carcasses, while group E contains only isolates of *Pseudomonas* from cuts obtained from slaughterhouses. Finally, it is noteworthy that one single *Escherichia* strain isolated from whole chickens acquired from abattoirs could not be grouped into any cluster.

## 3. Discussion

### 3.1. Identification of Colonies with Characteristic Morphology of Salmonella

The prevalence of Enterobacteriaceae recorded in the study being reported here, at 28.2% of the samples, is much lower than that noted by other authors investigating poultry meat [5,14] and further types of meat products [15]; research works that revealed the presence of these microorganisms in virtually 100% of the samples analyzed. These differences are due, at least in part, to the type of method used for the isolation of colonies, since in the present study the methodology concentrated on the isolation of *Salmonella* spp. and not enterobacteria in general. On the other hand, the results of the present investigation are consistent with those observed by other authors who indicate that the genera *Escherichia*, *Klebsiella*, *Pseudomonas*, *Proteus* or *Hafnia* are among those most frequently isolated from poultry meat [5,15]. The prevalence of genera found was quite similar for the two types of samples acquired in slaughterhouses, whole carcasses and cuts, but was very different from what was seen in meat preparations and cuts obtained from butcher shops. However, the higher overall prevalence of Enterobacteriaceae recorded in samples of carcasses (61.5%) and cuts from abattoirs (42.5%), as compared to cuts and preparations from butcher’s shops (13.1%) may be since these microorganisms are easily transmitted between carcasses during the process of plucking and eviscerating the birds [16].

In the present study, 93.0% of the strains with a typical *Salmonella* morphology isolated from the chromogenic medium turned out to be false positives after identification with MALDI-TOF. Some authors have also isolated *Klebsiella* strains in differential medium for *Salmonella* spp., although with a very different morphology and appearance, since they formed green or bluish colonies [17]. Using the ISO 6579:2017 standard, Evangelopoulou et al. [18] were able to isolate and identify 18 colonies with the typical morphology and appearance of *Salmonella* spp. on different specific culture media for the detection of this bacterium that after identification proved to be of the genera *Escherichia*, *Citrobacter*, *Trabulsiella* and *Klebsiella*. For their part, Pławińska-Czarnak et al. [19] detected 14.3% of strains with an apparent *Salmonella* identity that in fact corresponded to *Citrobacter braakii* and 12.4% that were really *Proteus mirabilis*. These results suggest that the effectiveness of the methods used for the detection and isolation of *Salmonella* spp. in poultry meat and poultry by-products are strongly influenced by the type of culture medium used, there being some with which better results are obtained than with others [20,21,22]. In the present study, some modifications were introduced with respect to the ISO 6579-1:2017 standard, which could have influenced the high percentage of false-positive results. Thus, only one secondary enrichment broth (Rappaport–Vasiliadis with soy) was used. According to ISO standard, selective enrichment for analyses of food samples must be performed in MKTTn broth and in Rappaport–Vassiliadis medium with soy or on MSRV agar. On the other hand, the isolation was carried out in one chromogenic medium (*Salmonella* Chromogen Agar Set), while two selective isolation agar media (XLD agar and a second medium for choice) must be used according to the ISO standard. In addition, according to ISO 6579-1:2017, confirmation shall be performed by biochemical (TSI, Urea, LDC) and serological testing. In the research being reported here MALDI-TOF is used for identification. These facts mean that the results obtained do not allow for evaluating the selectivity of the ISO standard, but rather the selectivity of the methodology used in this work. However, the variety of Enterobacteriaceae strains that have been isolated from culture media specifically designed for the isolation of *Salmonella*, both in the present study and in the published research that was consulted, suggest that it is necessary to develop better protocols for the detection of *Salmonella* in foods, with the aim of reducing the presence of false positives and allowing the various genera of Enterobacteriaceae to be distinguished with greater precision.

### 3.2. Antibiotic Susceptibility

The levels of resistance found in Enterobacteriaceae and *Pseudomonas*, accounting for 37.7% of all analyses performed and 36.2% of analyses performed for beta-lactam antibiotics, are high. Some authors have underlined the fact that resistance to antibiotics, and especially to the beta-lactam type, is increasingly determined by the mobilization of resistance genes. This transfer occurs through vehicles, for instance conjugative plasmids, that allow dissemination between species, provided that the donor and recipient bacteria are ecologically linked through a shared habitat, such as the gastrointestinal tract [23].

Most of the strains studied showed susceptibility to AK, FOX, IPM, CT, CN, MEM, TZP, C, FOS and TGC; results like those obtained by other authors for *Klebsiella pneumoniae* strains susceptible to AK, CN, CT and TGC [24] or *Escherichia coli* strains susceptible to AK, IPM and CIP [25]. In contrast, in the present study it was found that the majority of Enterobacteriaceae and *Pseudomonas* strains showed resistance or reduced susceptibility to several widely used antibiotics, such as TEC, E, RD, P, N and TEM. Other authors observed that the highest levels of resistance in strains of *E. coli* were to antibiotics such as AMC, FOX, CAZ or E [25].

In previous studies carried out on Enterobacteriaceae isolated from inert surfaces of poultry slaughterhouses, strains strongly susceptible (close to 100%) to CAZ and SXT were isolated, while the highest percentages of resistance for this microbial group were recorded for the antibiotics AMP, NA, S and MEM [26]. Comparing the results recorded in the present study with those in other published research showed striking differences between outcomes for the same substances [16,27,28,29,30]. These differences between investigations may be due to the differing origins of isolates, taken from slaughterhouse surfaces, different types of meat, clinical samples and so forth, or to the different bacterial genera considered whether *Hafnia*, *Escherichia*, *Klebsiella*, *Proteus* or *Pseudomonas*.

Scrutiny of the data based on the type of sample involved shows that in the present study the lowest percentages of resistance were observed in the samples from butcher’s shops, whilst in the carcasses and cuts obtained directly from slaughterhouses the resistance levels were considerably higher. These samples may have been cross-contaminated with persistent bacteria from abattoir processing environments. Such bacteria are exposed to constant cleaning and disinfection processes using a range of different antimicrobial products, a fact that has been associated with cross-resistance to various commonly used antibiotics [31]. It is striking that the high levels of resistance observed in strains from slaughterhouses were not found in those obtained from butcher’s shops, although this fact could be due to the different origins of the samples. It is unknown whether those acquired in shops came from the same abattoirs that provided material for study or, on the contrary, were processed in other slaughterhouses where different cleaning and disinfection protocols are applied.

Consideration of results by each genus of bacteria revealed differences for certain substances trialed, with strains of the genus *Hafnia* generally registering the lowest percentages of resistance. Other authors, however, found the highest percentages of resistance to SXT, SAM, CTX, FEP, KZ, IMP and MEM in strains of this microorganism [32]. On the other hand, the large percentages of resistance recorded in strains of *Klebsiella* and *Escherichia* agree with the data obtained by other researchers, who point out that resistance to antibiotics, and especially to beta-lactams, is very common in these genera [23].

### 3.3. Antibiotic Resistance Patterns

All the strains isolated, regardless of the origin of the sample or the bacterial genus to which they belonged, were resistant to multiple antibiotics, between 4 and 29 out of the 42 evaluated. As occurred with the percentages of resistance to each antibiotic, the strains isolated from carcasses presented the largest number of resistances per strain, followed by the joints obtained from slaughterhouses and, finally, joints and meat preparations from butcher’s shops. Scrutiny of these data at the genus level shows that *Klebsiella*, *Proteus* and *Pseudomonas* were the genera presenting the greatest number of resistances per isolate. If this information is taken together with figures for the prevalence of the various microbial groups, cuts and preparations from shops emerge as the group with the lowest number of resistances per isolate, since in these samples only colonies of the genera *Hafnia* and *Escherichia* were isolated, with the two genera showing the lowest resistance levels observed in the whole study.

The number of resistances per isolate was very variable. However, the figures indicate that all the bacteria presented an MDR phenotype, except for one strain of *Pseudomonas*, which showed an XDR phenotype. These data coincide with those observed by other authors, since the isolation of Enterobacteriaceae strains with the MDR phenotype from samples of poultry meat and derivatives, or from their processing environments, is increasingly common. This phenotype is also very widespread among bacteria of the genera *Klebsiella* [33,34,35,36], *Proteus* [37,38,39] or *Pseudomonas* [40,41,42], isolated from poultry meat, from live birds or from human clinical samples.

The outcome of a comparison of antibiotic resistance profiles, as shown in the tree diagram, indicates a clear relationship between strains of the same genus of bacteria, but an even stronger link between those that share the same habitat. This becomes evident from the observation that, of the five different groupings established by analysis, four were made up of strains isolated from the same type of samples (two for carcasses from slaughterhouses and another two for cuts from this same source), whilst only one of the clusters includes strains from more than one type of sample. Amaya et al. [43] also found different antibiotic resistance profiles dependent on the origin of *E. coli* strains isolated from wastewater. The exchange of resistance genes that can occur between these bacteria, a priori commensals and potentially pathogenic bacteria, with which they share a habitat, is worrying. It is thus especially crucial to perform constant epidemiological monitoring throughout the entire food chain, in order to reduce the risk for the consumer [44].

## 4. Materials and Methods

### 4.1. Sample Processing

The 234 samples used for analysis were whole chicken carcasses acquired directly from slaughterhouses (52 samples), chicken cuts also obtained from abattoirs (52) and chicken cuts and chicken preparations taken from butcher’s shops (130). All these establishments were in north-western Spain. To perform isolations, the method described in the ISO 6579-1:2017 standard [7] was followed, with some modifications. One selective broth (for secondary enrichment) and one selective agar (instead of two, as indicated in the ISO standard) were used in this research work. In addition, identification was performed by matrix-assisted laser desorption ionization and time-of-flight mass spectrometry (MALDI-TOF), while the ISO standard recommends biochemical and serological tests.

Twenty-five grams of skin from each sample were homogenized in 225 mL of buffered peptone water (Oxoid Ltd., Hampshire, UK) for two minutes, using a Masticator Silver (IUL Instruments, Barcelona, Spain). Bags containing the resultant homogenate were incubated at 37 °C for 24 h (primary enrichment). Thereafter, 100 μL of the homogenate were transferred to tubes with 10 mL of Rappaport–Vassiliadis broth (Oxoid) and incubated at 42 °C for a further 24 h (secondary enrichment). Finally, plates with selective differential medium (*Salmonella* Chromogen Agar Set, Sigma-Aldrich, St. Louis, MO, USA) were streaked with the Rappaport–Vassiliadis broth to detect *Salmonella* spp. The solid medium was incubated for 48 h at 37 °C.

### 4.2. Isolation and Identification of Strains

Colonies with the characteristic morphology and color of *Salmonella* spp. were isolated from the chromogenic culture medium, one being taken from each positive sample. Positive controls (plates with *Salmonella*) were used to determine the morphology of this microorganism on *Salmonella* Chromogen Agar Set and to compare the colonies with those grown from each sample. The selected items were preserved at −30 °C in TSB (Tryptone Soy Broth, Oxoid, Hampshire, UK) with 20% glycerol as a cryoprotectant. Identification was carried out using matrix-assisted laser desorption ionization and time-of-flight mass spectrometry (MALDI-TOF), following a methodology described in previous work [26]. Spectra were acquired automatically with the MALDI Biotyper system and, to perform a taxonomic assignment of each colony, they were compared with the reference database provided by Bruker Daltonics (Bremen, Germany). Match values above 2.0 were considered reliable for identification down to the genus level.

### 4.3. Antibiotic Resistance Determination

Antibiograms were performed with the strains isolated from the chromogenic medium *Salmonella* Chromogen Agar Set but not identified as *Salmonella* (“false positives”). Susceptibility to 42 antibiotics of clinical importance, in both human and veterinary medicine, was determined using the disc diffusion method [45]. To do this, the strains were incubated in 9 mL of Muller–Hinton Broth (MHB, Oxoid) at 37 °C until they reached the exponential growth phase, after approximately 6 hours. With these cultures, Muller–Hinton Agar plates (MHA, Oxoid) were inoculated using a sterile cotton swab, the selected antibiotic discs then being placed in position. The plates were incubated at 37 °C for 18-24 h, after which the inhibition zones were measured, with the strains being classified as susceptible, with reduced susceptibility (intermediate), or resistant, based on recommendations for Enterobacteriaceae and *Pseudomonas* [45,46].

The discs used contained antibiotics belonging to 13 different categories, including aminoglycosides: amikacin (AK, 30 μg), gentamycin (CN, 10 μg), kanamycin, (K, 30 μg), neomycin (N, 30 μg), streptomycin (S, 10 μg), spectinomycin (SH, 100 μg) and tobramycin (TOB, 10 μg); rifamycins: rifampicin (RD, 5 μg); beta-lactam antibiotics: amoxicillin/clavulanic acid (AMC, 30 μg), ampicillin/sulbactam (SAM, 20 μg), ampicillin (AMP, 10 μg), penicillin G (P, 10 μg), temocillin (TEM, 30 μg), ticarcillin (TIC, 75 μg), piperacillin/tazobactam (TZP, 110 μg), ceftazidime (CAZ, 30 μg), cefotaxime (CTX, 30 μg), cefepime (FEP, 30 μg), cefoxitin (FOX, 30 μg), cephalothin (KF, 30 μg), cephazolin (KZ, 30 μg), ertapenem (ETP, 10 μg), imipenem (IPM, 10 μg), meropenem (MEM, 10 μg) and aztreonam (ATM, 30 μg); phenicols: chloramphenicol (C, 30 μg); phosphomycins: phosphomycin (FOS, 50 μg); glycopeptides: teicoplanin (TEC, 30 μg); glycylglycines: tigecycline (TGC, 15 μg); macrolides: azithromycin (AZM, 15 μg) and erythromycin (E, 15 μg); nitrofurans: nitrofurantoin (F, 300 μg) and furazolidone (FR, 100 μg); polymyxins: colistin (CT, 10 μg); quinolones: ciprofloxacin (CIP, 5 μg), enrofloxacin (ENR, 5 μg) and nalidixic acid (NA, 30 μg); sulphonamides: sulphonamide (S3, 300 μg), sulphamethoxazole (RL, 25 μg), sulphamethoxazole/trimethoprim (SXT, 25 μg) and trimethoprim (W, 5 μg); and tetracyclines: tetracycline (TE, 30 μg).

### 4.4. Antibiotic Resistance Patterns

To categorize the antibiotic resistance profiles shown by each of the isolates, the criteria described by Magiorakos et al. [47] were used. These establish a multidrug-resistant (MDR) phenotype, indicating no susceptibility to at least one antibiotic from three or more antimicrobial categories. There is an extensively drug-resistant (XDR) phenotype, implying non-susceptibility to at least one agent in all but two or fewer antimicrobial categories. Finally, there is a pan-drug-resistant (PDR) phenotype, defined as lacking susceptibility to any of the antibiotics from all the categories tested.

### 4.5. Statistical Analysis

All statistical analyses were performed using RStudio software [48]. The prevalence of isolated Enterobacteriaceae and the percentages of antibiotic resistance were both analyzed with exact Chi-square tests, with a probability level of 5.0% (*p* < 0.05) being set for significant differences. Each resistance profile was classified by calculating the presence (1) or absence (0) of resistance for each antibiotic studied, similarities between resistance profiles being established with Euclidean distance coefficient. Finally, a tree diagram was drawn up using the full algorithm with the SRPlot tool [49].

## 5. Conclusions

The results obtained suggest that the method evaluated for the detection of *Salmonella* in food may not be the most appropriate procedure for the analysis of chicken meat samples. The culture media used for the isolation of *Salmonella* in the present work allowed the presence of a high percentage of false-positive results, consisting of other Enterobacteriaceae (*Hafnia*, *Escherichia*, *Klebsiella* and *Proteus*) and *Pseudomonas*, whose colonies have a similar morphology and color to those of *Salmonella*. This can cause errors in the detection of *Salmonella* and makes the full identification of any strains isolated essential. Furthermore, the high levels of resistance to commonly used antibiotics that were found, together with the wide prevalence of isolates with the MDR phenotype, indicate that Enterobacteriaceae and *Pseudomonas* isolates from poultry meat and its derived products are important reservoirs of resistant bacteria, implying a potential risk for consumers’ health. These findings point to a need to improve antibiotic resistance monitoring protocols to minimize the presence of antibiotic-resistant bacteria in poultry.

## Figures and Tables

**Table 1:** Average number of resistances per isolate and percentages of resistance, reduced susceptibility or susceptibility. The data are shown separately by type of sample and by genus.

Sample Type/Microbial Genera	Resistance *		Reduced Susceptibility *		Susceptibility *	
	Number ATB	%	Number ATB	%	Number ATB	%
**Carcasses ^1^**	18.89 ± 7.13	45.0	3.91 ± 1.54	9.3	19.19 ± 5.84	45.7
**Cuts ^2^**	15.81 ± 7.13	37.6	5.19 ± 2.30	12.4	12.37 ± 6.93	50.0
**Cuts and Preparations ^3^**	7.75 ± 2.38	18.5	4.25 ± 2.09	10.1	30.00 ± 3.50	71.4
***Hafnia***	7.33 ± 1.15	17.5	2.33 ± 1.53	5.6	32.33 ± 0.58	77.0
***Escherichia***	13.41 ± 7.31	31.9	4.27 ± 4.23	10.2	24.32 ± 7.09	57.9
***Klebsiella***	17.89 ± 4.63	42.6	3.42 ± 1.30	8.1	20.68 ± 4.32	49.2
***Proteus***	19.17 ± 4.79	45.6	4.17 ± 2.14	9.9	18.67 ± 4.97	44.4
***Pseudomonas***	17.06 ± 8.39	40.6	6.44 ± 2.25	15.3	18.50 ± 7.19	44.0

* The percentages have been calculated based on the number of reactions showing resistance, reduced susceptibility or susceptibility relative to the total number of determinations (42 antibiotics × number of strains in each case). ATB, antibiotics. ^1^ Chicken carcasses obtained from slaughterhouses, ^2^ chicken cuts from slaughterhouses, ^3^ chicken cuts or chicken preparations acquired from butcher’s shops.

## Data Availability

The data presented in this study are available on request from the corresponding author.

## References

[1] Farmer J.J., Farmer M.K., Holmes B., Borriello S.P., Murray P.R., Funke G. (2010). The Enterobacteriaceae: General characteristics. Topley & Wilson’s Microbiology and Microbial Infections.

[2] Zeng H., De Reu K., Gabriël S., Mattheus W., De Zutter L., Rasschaert G. (2021). *Salmonella* prevalence and persistence in industrialized poultry slaughterhouses. Poult. Sci..

[3] Buzón-Durán L., Capita R., Alonso-Calleja C. (2017). Microbial loads and antibiotic resistance patterns of *Staphylococcus aureus* in different types of raw poultry-based meat preparations. Poult. Sci..

[4] OECD-FAO Agricultural Outlook 2023–2032. https://www.oecd-ilibrary.org/agriculture-and-food/oecd-fao-agricultural-outlook-2023-2032_08801ab7-en.

[5] Capita R., Castaño-Arriba A., Rodríguez-Melcón C., Igrejas G., Poeta P., Alonso-Calleja C. (2020). Diversity, antibiotic resistance, and biofilm-forming ability of enterobacteria isolated from red meat and poultry preparations. Microorganisms.

[6] Lee K.M., Runyon M., Herrman T.J., Phillips R., Hsieh J. (2015). Review of *Salmonella* detection and identification methods: Aspects of rapid emergency response and food safety. Food Control.

[7] (2017). Microbiology of the Food Chain—Horizontal Method for the Detection, Enumeration and Serotyping of *Salmonella*—Part 1: Detection of *Salmonella* spp..

[8] Lauer W.F., Martinez F.L., Hammack T. (2009). RAPID’ *Salmonella* Chromogenic Medium. J. AOAC Int..

[9] Bruzzese E., Giannattasio A., Guarino A. (2018). Antibiotic treatment of acute gastroenteritis in children. Food Res..

[10] Rozwandowicz M., Brouwer M.S.M., Fischer J., Wagenaar J.A., Gonzalez-Zorn B., Guerra B., Mevius D.J., Hordijk J. (2018). Plasmids carrying antimicrobial resistance genes in Enterobacteriaceae. J. Antimicrob. Chemother..

[11] Morrill H.J., Pogue J.M., Kaye K.S., LaPlante K.L. (2015). Treatment options for carbapenem-resistant *Enterobacteriaceae* infections. Open Forum Infect. Dis..

[12] Miranda C., Silva V., Capita R., Alonso-Calleja C., Igrejas G., Poeta P. (2020). Implications of antibiotics use during the COVID-19 pandemic: Present and future. J. Antimicrob. Chemother..

[13] Capita R., Alonso-Calleja C. (2013). Antibiotic-resistant bacteria: A challenge for the food industry. Crit. Rev. Food Sci. Nutr..

[14] Díaz-Jiménez D., García-Meniño I., Fernández J., García V., Mora A. (2020). Chicken and turkey meat: Consumer exposure to multidrug-resistant Enterobacteriaceae including *mcr*-carriers, uropathogenic *E. coli* and high-risk lineages such as ST131. Int. J. Food Microbiol..

[15] Uzeh R.E., Adewumi F., Odumosu B.T. (2021). Antibiotic resistance and plasmid analysis of Enterobacteriaceae isolated from retail meat in Lagos Nigeria. One Health Outlook.

[16] Gregová G., Kmetova M., Kmet V., Venglovsky J., Feher A. (2012). Antibiotic resistance of *Escherichia coli* isolated from a poultry slaughterhouse. Ann. Agric. Environ. Med..

[17] Manal A.H., Saad S.F., Zahraa A.J., Saba T.H. (2015). Chromogenic agar media for rapid detection of *Enterobacteriaceae* in food samples. Afr. J. Microbiol. Res..

[18] Evangelopoulou G., Burriel A.R., Solomakos N. (2024). Distinctive culture expressions of enterobacteria interfering with isolation of *Salmonella* spp. during the application of the recommended ISO 6579-1: 2017. Appl. Sci..

[19] Pławińska-Czarnak J., Wódz K., Kizerwetter-Świda M., Nowak T., Bogdan J., Kwieciński P., Kwieciński A., Anusz K. (2021). *Citrobacter braakii* yield false-positive identification as *Salmonella*, a note of caution. Foods.

[20] Carrique-Mas J.J., Barnes S., McLaren I., Davies R. (2009). Comparison of three plating media for the isolation of *Salmonella* from poultry environmental samples in Great Britain using ISO 6579: 2002 (Annex D). J. Appl. Microbiol..

[21] Love B.C., Rostagno M.H. (2008). Comparison of five culture methods for *Salmonella* isolation from swine fecal samples of known infection status. J. Vet. Diagn. Investig..

[22] Yhiler N.Y., Bassey E.B., Useh M.F. (2015). Evaluation of the performance of two selective enrichment media and two selective plating media for the detection of *Salmonella* from primary poultry production, according to ISO 6579: 2002. Open J. Med. Microbiol..

[23] Iredell J., Brown J., Tagg K. (2016). Antibiotic resistance in Enterobacteriaceae: Mechanisms and clinical implications. BMJ.

[24] Akgül Ö., Körkoca H., Bora G. (2024). Analysis of antibiotic resistance of KPC-2-positive *Klebsiella pneumoniae* strains isolated from blood cultures in Van, Turkey. Eur. Rev. Med. Pharmacol. Sci..

[25] Nada H.G., El-Tahan A.S., El-Didamony G., Askora A. (2023). Detection of multidrug-resistant Shiga toxin-producing *Escherichia coli* in some food products and cattle feces in Al-Sharkia, Egypt: One health menace. BMC Microbiol..

[26] Panera-Martínez S., Rodríguez-Melcón C., Rodríguez-Campos D., Pérez-Estébanez N., Capita R., Alonso-Calleja C. (2024). Levels of different microbial groups on inert surfaces of poultry slaughterhouses: Identification using matrix-assisted laser desorption ionization time-of-flight and detection of extended-spectrum beta-lactamase-and carbapenemase-producing Enterobacteria. Antibiotics.

[27] Barka M.S., Cherif-Anntar A., Benamar I. (2021). Antimicrobial resistance patterns and transferable traits in Enterobacteriaceae isolates from poultry in Tlemcen, Algeria. Afr. J. Clin. Experimental Microbiol..

[28] Bushen A., Tekalign E., Abayneh M. (2021). Drug-and multidrug-resistance pattern of Enterobacteriaceae isolated from droppings of healthy chickens on a poultry farm in Southwest Ethiopia. Infect. Drug Res..

[29] Elabbasy M.T., Hussein M.A., Algahtani F.D., Abd El-Rahman G.I., Morshdy A.E., Elkafrawy I.A., Adeboye A.A. (2021). MALDI-TOF MS based typing for rapid screening of multiple antibiotic resistance *E. coli* and virulent non-O157 shiga toxin-producing *E. coli* isolated from the slaughterhouse settings and beef carcasses. Foods.

[30] Savin M., Bierbaum G., Mutters N.T., Schmithausen R.M., Kreyenschmidt J., García-Meniño I., Schmoger S., Käsbohrer A., Hammerl J.A. (2022). Genetic characterization of carbapenem-resistant *Klebsiella* spp. from municipal and slaughterhouse wastewater. Antibiotics.

[31] Puangseree J., Jeamsripong S., Prathan R., Pungpian C., Chuanchuen R. (2021). Resistance to widely-used disinfectants and heavy metals and cross resistance to antibiotics in *Escherichia coli* isolated from pigs, pork and pig carcass. Food Control.

[32] Rizi K.S., Hasanzade S., Soleimanpour S., Youssefi M., Jamehdar S.A., Ghazvini K., Safdari H., Farsiani H. (2021). Phenotypic and molecular characterization of antimicrobial resistance in clinical species of *Enterobacter*, *Serratia*, and *Hafnia* in Northeast Iran. Gene Rep..

[33] Elmonir W., Abd El-Aziz N.K., Tartor Y.H., Moustafa S.M., Abo Remela E.M., Eissa R., Saad H.A., Tawab A.A. (2021). Emergence of colistin and carbapenem resistance in extended-spectrum β-lactamase producing *Klebsiella pneumoniae* isolated from chickens and humans in Egypt. Biology.

[34] Li Z., Xin L., Peng C., Liu C., Wang P., Yu L., Liu M., Wang F. (2022). Prevalence and antimicrobial susceptibility profiles of ESBL-producing *Klebsiella pneumoniae* from broiler chicken farms in Shandong Province, China. Poult. Sci..

[35] Permatasari D.A., Witaningrum A.M., Wibisono F.J., Effendi M.H. (2000). Detection and prevalence of multidrug-resistant *Klebsiella pneumoniae* strains isolated from poultry farms in Blitar, Indonesia. Biodivers. J. Biol. Divers..

[36] Veloo Y., Thahir S.S., Rajendiran S., Hock L.K., Ahmad N., Muthu V., Shaharudin R. (2022). Multidrug-resistant gram-negative bacteria and extended-spectrum β-lactamase-producing *Klebsiella pneumoniae* from the poultry farm environment. Microbiol. Spectr..

[37] Ishaq K., Ahmad A., Rafique A., Aslam R., Ali S., Shahid M.A., Sarwar N., Aslam M.A., Aslam B., Arshad M.I. (2022). Occurrence and antimicrobial susceptibility of *Proteus mirabilis* from chicken carcass. Pak. Vet. J..

[38] Li Z., Peng C., Zhang G., Shen Y., Zhang Y., Liu C., Liu M., Wang F. (2022). Prevalence and characteristics of multidrug-resistant *Proteus mirabilis* from broiler farms in Shandong Province, China. Poult. Sci..

[39] Owoseni M.C., Oyigye O., Sani B., Lamin J., Chere A. (2021). Antimicrobial resistance and virulence genes profiling of *Proteus* species from poultry farms in Lafia, Nigeria. bioRxiv.

[40] Abd El-Ghany W.A. (2021). *Pseudomonas aeruginosa* infection of avian origin: Zoonosis and one health implications. Vet. World.

[41] Marouf S., Li X., Salem H.M., Ahmed Z.S., Nader S.M., Shaalan M., Awad F.H., Zhou H., Cheang T. (2023). Molecular detection of multidrug-resistant *Pseudomonas aeruginosa* of different avian sources with pathogenicity testing and in vitro evaluation of antibacterial efficacy of silver nanoparticles against multidrug-resistant *P. aeruginosa*. Poult. Sci..

[42] Odoi H., Boamah V.E., Boakye Y.D., Agyare C. (2021). Prevalence and phenotypic and genotypic resistance mechanisms of multidrug-resistant *Pseudomonas aeruginosa* strains isolated from clinical, environmental, and poultry litter samples from the Ashanti region of Ghana. J. Environ. Public Health.

[43] Amaya E., Reyes D., Paniagua M., Calderón S., Rashid M.U., Colque P., Kühn I., Möllby R., Weintraub A., Nord C.E. (2012). Antibiotic resistance patterns of *Escherichia coli* isolates from different aquatic environmental sources in Leon, Nicaragua. Clin. Microbiol. Infect..

[44] Costa R.G., Festivo M.L., Araujo M.S., Reis E.M., Lázaro N.S., Rodrigues D.P. (2013). Antimicrobial susceptibility and serovars of *Salmonella* circulating in commercial poultry carcasses and poultry products in Brazil. J. Food Prot..

[45] CLSI (2023). Performance Standards for Antimicrobial Susceptibility Testing.

[46] EUCAST. The European Committee on Antimicrobial Susceptibility Testing. Breakpoint Tables for Interpretation of MICs and Zone Diameters. Version 13.1. http://www.eucast.org.

[47] Magiorakos A.P., Srinivasan A., Carey R.B., Carmeli Y., Falagas M.E., Giske C.G., Harbarth S., Hindler J.F., Kahlmeter G., Olsson-Liljequist B. (2012). Multidrug-resistant, extensively drug-resistant and pandrug-resistant bacteria: An international expert proposal for interim standard definitions for acquired resistance. Clin. Microbiol. Infect..

[48] RStudio Team RStudio: Integrated Development Environment for R. http://www.rstudio.com/.

[49] Tang D., Chen M., Huang X., Zhang G., Zeng L., Zhang G., Wu S., Wang Y. (2023). Rplot: A free online platform for data visualization and graphing. PLoS ONE.

